# Research progress of neonatal hypoxic‐ischemic encephalopathy in nonhuman primate models

**DOI:** 10.1002/ibra.12097

**Published:** 2023-03-26

**Authors:** Yi‐Huan Guan, Hong‐Su Zhou, Bo‐Yan Luo, Sajid Hussain, Liu‐Lin Xiong

**Affiliations:** ^1^ School of Anesthesiology Zunyi Medical University Zunyi China; ^2^ Department of Experimental Animals Kunming Medical University Kunming China; ^3^ School of Pharmacy Zunyi Medical University Zunyi China; ^4^ NUTECH School of Applied Sciences and Humanities National University of Technology Islamabad Pakistan; ^5^ School of Pharmacy and Medical Sciences, Faculty of Health Sciences University of South Australia Adelaide South Australia Australia

**Keywords:** biomarkers, ischemic and hypoxic encephalopathy, nonhuman primate, pathology, treatment

## Abstract

Neonatal hypoxic‐ischemic encephalopathy (HIE) is one of the important complications of neonatal asphyxia, which not only leads to neurological disability but also seriously threatens the life of neonates. Over the years, animal models of HIE have been a research hotspot to find ways to cope with HIE and thereby reduce the risk of neonatal death or disability in moderate‐to‐severe HIE. By reviewing the literature related to HIE over the years, it was found that nonhuman primates share a high degree of homology with human gross neural anatomy. The basic data on nonhuman primates are not yet complete, so it is urgent to mine and develop new nonhuman primate model data. In recent years, the research on nonhuman primate HIE models has been gradually enriched and the content is more novel. Therefore, the purpose of this review is to further summarize the methods for establishing the nonhuman primate HIE model and to better elucidate the relevance of the nonhuman primate model to humans by observing the behavioral manifestations, neuropathology, and a series of biomarkers of HIE in primates HIE. Finally, the most popular and desirable treatments studied in nonhuman primate models in the past 5 years are summarized.

## Graphical abstract

Hypoxic‐ischemic encephalopathy (HIE) models in nonhuman primates are mainly constructed by umbilical cord occlusion (UCO), acute total asphyxia, and partial asphyxia. The latter two of these are more novel than UCO, and the clinical manifestations and brain histopathology of these two models also differ because they preferentially injure different parts of the brain. In addition, many studies have shown that nonhuman primates are highly similar to humans in terms of brain injury patterns and development. A series of important chemical indicators exist in nonhuman primate HIE models that can be used as diagnostic and/or prognostic indicators of HIE. Finally, we further summarize the treatments evaluated in nonhuman primate models in recent years and conclude that therapeutic hypothermia (TH) + erythropoietin (EPO) is currently the most effective treatment in animal models.

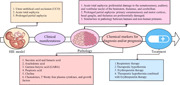

## 1 INTRODUCTION

Neonatal hypoxic‐ischemic encephalopathy (HIE) refers to hypoxic‐ischemic damage to the brain caused by perinatal asphyxia, including characteristic neuropathological and pathophysiological changes and a series of clinical manifestations of encephalopathy. Severe hypoxic‐ischemic in neonates results in complex physiological, cellular, and molecular changes that further lead to premature death or acute symptoms including altered consciousness, seizures, weak breathing, metabolic disturbances or poor muscle tone, and chronic diseases such as epilepsy, cerebral palsy (CP), intellectual disability and mobility impairment.^1^, ^2^ This disease is one of the important diseases in the neonatal period, with severe illness and high mortality. In severe cases, permanent neurological deficits can occur, affecting the survival and quality of life of newborns. The incidence of HIE is approximately 1.5 per 1000 live births in developed countries and 10%–20% per 1000 live births in low‐ and middle‐income countries.^3^, ^4^, ^5^ Nonhuman primate models have significant brain tissue damage involving neuronal degeneration and cytologic necrotizing.^6^ If the apoptotic process can be inhibited and blocked by intervention, and delayed necrosis of nerve cells can be effectively prevented, it is possible to alleviate the brain damage caused by hypoxia‐ischemia in neonates.^6^, ^7^ Therapeutic hypothermia (TH) is the standard of care and is currently the most commonly used treatment to reduce the risk of death or disability in newborns with moderate to severe HIE. Hypothermia treatment begins within 6 h of birth, which is optimal timing, and lasts 72 h.^1^ According to research, TH can reduce brain damage by inhibiting the excito‐oxidative cascade, but it does not provide complete neuroprotection, and even after treatment, the mortality rate and the incidence of neurological encephalopathy remain high.^8^ This is one of the reasons why so many scholars are still devoted to the study of neonatal HIE. To study HIE, humans have established many animal models. In general, rodent (mouse and rat) models can provide greater flexibility and are more cost‐effective to test drug efficacy in a dose–response manner. Large animal models, including piglets, sheep, and nonhuman primates, can be used as a third step for more focused and accurate translational studies, including pharmacokinetic and safety pharmacologic assessments.^9^ Therefore, this review aims to derive the best animal model of HIE by comparing pre‐existing models of HIE. By observing the behavioral manifestations, neuropathology, and a series of biomarkers of HIE in nonhuman primates, the correlation between the nonhuman primate HIE model and humans is further clarified. Finally, the most popular and ideal treatments studied in nonhuman primate models in recent years are summarized.

## 2 SUMMARY OF HIE MODELS

### 2.1 Rodent model

Rodents are the most common model used in HIE studies. Their advantages include ease of manipulation and small size for testing, low cost, stable genetic features, and diverse behavioral features for neurobehavioral studies.^10^ But odontoid exhibits a lissencephalic brain with a paucity of cerebral white matter, which lacks the clear separability of cortical divisions similar to those of Brodman's area in humans. Moreover, the supply of cerebral vessels is significantly different from that of humans. There are major neuroanatomical drawbacks to the use of rodent models of HIE, which can be compensated for by larger animals (Table 1).^6^, ^11^.

**Table 1:** Summary of hypoxic‐ischemic encephalopathy (HIE) models.

Model	Disadvantages	Advantages
Rodents	1. Neuroanatomical drawbacks.	1. Ease of manipulation and small size for testing, low cost. 2. Stable genetic features and diverse behavioral features for neurobehavioral studies.
Piglet	1. The evolution of neurological and functional parameters and/or injury over time could not be followed. 2. The lack of homogeneous genetic background among individuals increases variability. 3. High cost.	1. The brain is similar in structure and maturity to that of a human newborn. 2. Body surface area and physiology are similar to those of human neonates.
Sheep	1. Huge costs and technical challenges. 2. The surgical trauma of pregnant sheep is large and the recovery time is long.	1. Brain development and neurodevelopment are similar to humans.
Nonhuman primate	1. Economic costs. 2. Ethical concerns. 3. Practical limitations.	1. It is highly similar to humans in terms of genetics, anatomy, and physiological function. 2. The brain is similar to the human brain in terms of complexity and development, with higher brain functions. 3. Behaving very similar to humans, also living in social groups, with complex and advanced cognitive processes.

### 2.2 Piglet model

The brains of newborn piglets are more similar in structure and maturity than those of rodents. The body surface area and physiology of piglets are comparable to those of human neonates, allowing more accurate prediction of pharmacokinetics and dose in humans.^12^ Experiments in the piglet model can be performed within 48–72 h after injury, so it is not possible to follow up on neurological and functional parameters and/or the evolution of injury over time. The lack of homogeneous genetic background among individuals enhances variability. At the same time, the piglet model is expensive, and the high cost is associated with a long time since usually only one or two animals can be handled in an experiment, thus limiting the number of subjects that can be included in the study (Table 1).^6^, ^9^

### 2.3 Sheep model

The neurodevelopment of sheep, a relatively large animal used in research, at 95 days of gestation is comparable to that of human oligodendrocyte (OL) development, the detection of auditory and somatosensory stimuli in the cerebral cortex, and the development of cerebral sulci at 24–28 weeks of gestation.^6^ However, the ovine fetal model is mostly a model of occlusive cerebral ischemia, which requires long‐term implantation of instruments.^13^ The sheep fetal model faces significant cost and technical challenges, including facilities specifically equipped with equipment and personnel, an experienced surgical team, and special care in the intensive care unit. Another point of consideration is the severe surgical trauma and long recovery time in pregnant sheep. Given the technical complexity of conducting such studies, the sheep model is hardly cost‐effective, thus limiting the availability of this model for drug discovery (Table 1).^6^, ^11^

### 2.4 Nonhuman primate models

From my perspective, the best choice for building a model is definitely a nonhuman primate model. Nonhuman primates are not only mediators between human and rodent models but also ideal experimental animals for the establishment of many psychiatric disease models due to their high similarity to humans in genetics, anatomical structure, and physiological function;^14^ Most importantly, highly similar in cellular structure to the human brain. Therefore, primates have been widely used in human neuroimaging, primate neurophysiology, developmental neuropsychological research, visual system research, viral disease, and tumor research.^15^, ^16^, ^17^ Interestingly, studies of the brain mechanisms underlying emotion in nonhuman primates and apes have reported the same emotional tonal representations in the human and ape brains, and this reasoning is supported by neurobehavioral models.^18^ Like humans, the prefrontal cortex and amygdala of nonhuman primates have the functions of regulating emotion and motivation, which makes their responses in the face of stress stimuli very similar to those of humans, including avoidance behavior, increased vigilance and arousal, activation of the sympathetic nervous system of the autonomic nervous system, and cortisol secretion by the adrenal gland.^19^ The brains of nonhuman primates are similar in complexity and development to the human brain, possess higher brain functions, and can be trained to perform specific types of test tasks. Therefore, compared with other animal models, it has an irreplaceable role in evaluating cognitive ability and emotional reaction.^20^ This facilitates neurocognitive testing over time, similar to testing in humans. Finally, nonhuman primates behave very similarly to humans, also live in social groups, and have complex, advanced cognitive processes.^21^ These properties make this utero umbilical cord occlusion (UCO) model attractive for studying the long‐term neuropathology of HIE‐related CP and the safety and efficacy of neurotherapeutics.^22^ However, nonhuman primates also have many limitations, including economic costs, ethical concerns, and practical limitations. Due to the high cost of the model, the number of animals in each group is small, which inevitably increases the error of the experimental data.^22^ Overall, this unique baboon model of prematurity is neuropathologically very similar to human prematurity. Although it has certain limitations in some aspects, it does not affect it becoming the preferred model (Table 1).

## 3 ESTABLISHMENT OF NONHUMAN PRIMATES HIE MODELS

### 3.1 UCO model

In 1959, Rank and Windle studied birth asphyxia in nonhuman primates. They delivered 5 (157–164 days of gestation, 166 days of gestation ¼) macaque fetuses (*rhesus macaques*) by hysterectomy, waited 11–16 min, then opened the amniotic sac and resuscitated with pulmonary oxygen to obtain a rhesus macaque HIE model.^6^, ^23^ In recent years, the UCO method was used to establish the HIE model: full‐term (173 days) or 9 ± 2 days before delivery, the nonhuman primate babies were delivered by cesarean section under general anesthesia. The umbilical cord was removed after the uterus was dissected, leaving the amniotic fluid and the fetus in the uterus. To simulate perinatal asphyxia, UCO was performed for about 18 min. During UCO, the fetal heart rate was monitored and all infusions and blood collections were allowed. After delivery, doctors use standardized principles of neonatal resuscitation to weigh and stabilize the fetus. Resuscitation measures include endotracheal intubation, positive pressure ventilation, chest compressions, and epinephrine as needed. Heating pads, radiant heaters, and polyethylene sheets provide thermal support.^24^, ^25^, ^26^

### 3.2 Acute total asphyxia model and prolonged partial asphyxia model

**Figure 1:**
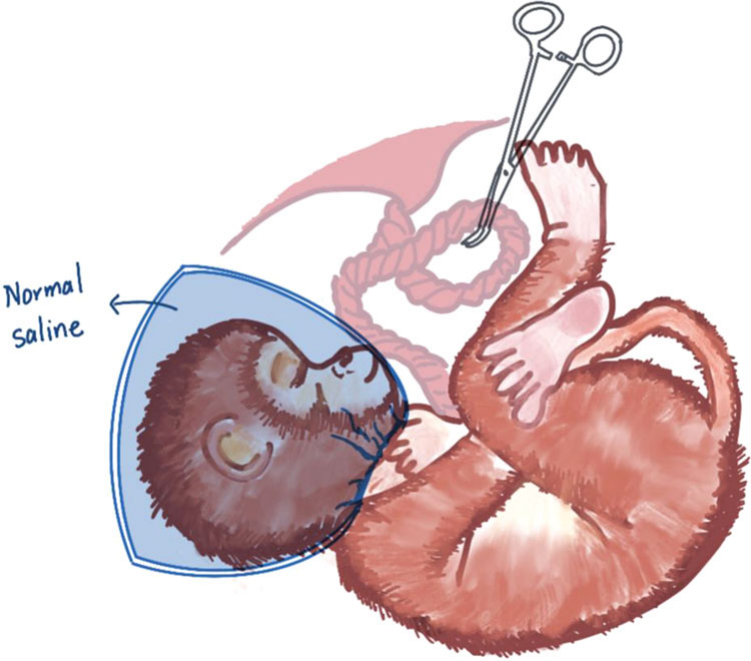
Preparation of acute total asphyxia model: It is formed by placing a thin, saline‐filled rubber bladder on the head of a full‐term nonhuman primate fetus during surgical delivery while clamping the umbilical cord. The wrapping of the fetal head prevents the initiation of breathing, and the clamping of the umbilical cord hinders the circulation of the placental blood between the fetus and the mother. [Color figure can be viewed at wileyonlinelibrary.com]

**Figure 2:**
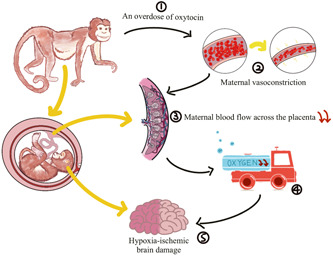
Preparation of prolonged partial asphyxia: ① An overdose of oxytocin is injected into the maternal blood. ② Excess oxytocin stimulates the mother to experience frequent, intense uterine contractions and drastic vasoconstriction. ③ Maternal vasoconstriction reduces blood flow to the uterus, thereby reducing maternal blood flow across the placenta. ④ Delayed perfusion of the placental intervillous space significantly reduced the net exchange of respiratory gases between the mother and fetus, resulting in a significant decrease in the oxygen content of the fetal blood. ⑤ This eventually leads to hypoxic‐ischemic brain damage in neonates. [Color figure can be viewed at wileyonlinelibrary.com]

On the basis of the UCO model, two models of acute total asphyxia and prolonged partial asphyxia were derived. Acute total asphyxia is formed by placing a thin, saline‐filled rubber sac over the fetal head of a full‐term nonhuman primate fetus during surgical delivery while clipping the umbilical cord. The wrapping of the fetal head blocks the initiation of respiration, and the clamping of the umbilical cord blocks the circulation of the placental blood between the fetus and the mother. Together these two measures result in a complete cessation of fetal respiratory gas exchange (Figure 1).^27^ On the other hand, the onset of prolonged partial asphyxia can be caused in a number of ways, the main mechanism being that the maternal blood flow through the placenta was somehow reduced. Ronald Myers's first experiment chose to inject excess oxytocin into the mother's blood. Excessive oxytocin stimulates the mother, causing it to experience frequent, strong uterine contractions and violent vasoconstriction.^27^ Fetal hypotension and bradycardia occur after uterine contractions. This hypotension may be closely related to cerebral perfusion, and repeated episodes may easily lead to fetal brain damage. Maternal vasoconstriction reduces blood flow to the uterus, thereby reducing maternal blood flow across the placenta. Delayed perfusion of the placental intervillous space significantly reduces the net exchange of respiratory gases between mother and fetus, resulting in varying degrees of hypoxia, acidosis, and hypercapnia (Figure 2).^27^

## 4 BEHAVIORAL MANIFESTATIONS OF NONHUMAN PRIMATE HIE MODELS

The behavioral manifestations of nonhuman primate HIE models are diverse. Some nonhuman primate fetuses developed severe pathological impairment of the descending motor pathway, manifested first by acute bilateral rigidity, followed by alternating weakness and rigidity.^28^, ^29^, ^30^, ^31^ They also exhibit decreased vestibulo‐ocular and pupillary light reflexes and oral motor dysfunction consistent with brainstem pathology and dorsal brainstem syndrome in human infants.^32^, ^33^ In addition, athetosis, inability to turn to the right, and status epilepticus were occasionally seen.^6^

## 5 PATHOLOGY OF NONHUMAN PRIMATE HIE MODELS

In the nonhuman primate HIE model, the common injury patterns are divided into total asphyxia and partial asphyxia. Among them, partial asphyxia is more common, because the pattern of brain injury caused by partial asphyxia is more similar to the typical injury pattern commonly seen in human brain tissue.^7^ Studies have shown that total asphyxia with persistently decreased arterial pressure produces neurological symptoms of CP with preferential damage to the somatosensory, auditory, and vestibular nuclei of the brainstem, thalamus, and cerebellum.^22^ However, in the partial asphyxia model, primary somatosensory and motor cortices, basal ganglia, and thalamus were preferentially damaged.^6^

### 5.1 Acute total asphyxia model

There is a small number of apparent neuronal deletions in the nonhuman primate dentate gyrus, but the bulk of the hippocampus appears normal, unlike the vulnerability of the neonatal hippocampus, especially after clinical seizures. Deletion of cerebellar Purkinje neurons was largely confined to the vermis. The cerebral cortical injury involves the degeneration of isolated pyramidal neurons that are cytologically necrotizing. In humans with HIE, cerebral and cerebellar cortical lesions are generally milder than subcortical lesions, but seizures are clearly associated with the amount of cortical necrosis.^6^

Nonhuman primates with HIE also showed white matter damage. Demyelination is seen in various white matter tracts including the internal capsule. Myelin degeneration appears to occur concurrently with axonal degeneration. Individual OLs in degenerative white matter show cytopathology. Leukoencephalopathy is particularly associated with human neonatal HIE, although earlier studies could not attribute perinatal leukoencephalopathy to a single etiology.^6^

### 5.2 Prolonged partial asphyxia model

Interestingly, in the partial injury model studied by Faro and Windle, the delayed injury in the hippocampus was minimal compared to the total asphyxia model, and progressive pathology in the putamen, cerebellum, and globus pallidus was not evident. Spinal dorsal horns and mesolateral columns showed atrophy, and brainstem pathology (especially in the cochlear nucleus) evolves. There was also preferential damage in the thalamus, where the anterior ventral, centromedial, and occipital nuclei showed secondary lesion prolongation. The paracentral gyrus and basal ganglia of the neocortex were selectively vulnerable, and the brainstem was less damaged. Compared with the acute total asphyxia model, the prolonged partial asphyxia model is closer to the typical injury model of human HIE.^6^, ^7^

### 5.3 Correlations and differences between nonhuman primate models and humans

According to brain histopathology, conventional magnetic resonance imaging (MRI), and diffusion MRI, preterm nonhuman primate models born at Days 125, 140, and 160 are equivalent to those born at 26–28 weeks, 30–32 weeks, and term, respectively. The most common neuropathological injury in nonhuman primate preterm infants, as in human preterm infants, is white matter damage, mostly in the parietal and occipital lobes. White matter damage is manifested by the activation of microglia and hyperplasia of reactive astrocytes, as well as increased ventricular volume. The second most common is the ventricular, germinal matrix, and subarachnoid hemorrhage. This injury is usually diffuse, and cystic infarcts rarely occur. These preliminary results support the model's high similarity to human preterm infant brain injury patterns and development.^34^, ^35^ Interestingly, Koehler et al. have reported that in human infants with HIE, some clinical manifestations such as decreased limb activity, hypotonia, and diminished or even absent deep tendon reflexes are most likely due to spinal cord injury.^6^ Moreover, basal ganglia damage was found in globus pallidus, the caudal half of the putamen, mirroring the damage seen in term human HIE.^36^, ^37^, ^38^ However, there are some differences between this model and human infants. In the study by Irene A M Schiering et al, strong aquaporin‐4 immunoreactivity was seen on astroglial cells within hippocampi in 50% of cases. The complex cellular and molecular cascades that occur in the hippocampus of human neonates after perinatal asphyxia are confirmed. These alterations may promote the development of epilepsy, leading to secondary brain damage.^39^ In primate fetuses, however, especially after clinical seizures, unlike the vulnerability of the human fetal hippocampus, most of the nonhuman primate's hippocampus is normal, with only minor neurological damage in the dentate gyrus. In nonhuman primate HIE, the loss of cerebellar Purkinje neurons is largely confined to the vermis. Damage to the cerebral cortex involves the degeneration of isolated pyramidal neurons with necrotizing cytology. In human HIE, cerebral and cerebellar cortical pathological lesions tend to be less severe than subcortical lesions, but the degree of seizures clearly correlates with the severity of cortical necrosis.^6^, ^39^ Overall, this unique primate HIE model is slightly different in neuropathology from human HIE, but the similarity is still greater (Table 2). However, there are also some issues, such as ethical issues, economic costs, and the complexity of nonhuman primate research, which limit the availability of this model for drug discovery. Despite these limitations, numerous studies have provided valuable insights into long‐term brain neuropathology associated with CP secondary to HIE, findings that cannot be determined in humans due to obvious ethical reasons and limitations of human postmortem studies, including postmortem delays and differences in underlying etiologies.^22^ We believe that selective studies of this model will provide additional insights into the etiology of hypoxic‐ischemic brain injury and altered brain development in humans.

**Table 2:** Correlations and differences between nonhuman primate models and humans.

Relationships species	Human	Nonhuman primate
Correlations	1. The most common neuropathological damage is white matter damage, mainly located in the parietal and occipital lobes. 2. The second most common are ventricular, germinal matrix, and subarachnoid hemorrhage. 3. Basal ganglia damage was found in globus pallidus, the caudal half of the putamen.	
Differences	1. Complex cellular and molecular cascades occurring in the hippocampus of human neonates after perinatal asphyxia.	1. Most of the nonhuman primate's hippocampus is normal, with only minor neurological damage in the dentate gyrus.
	2. Cerebral and cerebellar cortical pathological lesions tend to be less severe than subcortical lesions.	2. Marked lesions in the cerebral and cerebellar cortex.

## 6 BIOMARKERS OF NONHUMAN PRIMATE HIE MODELS

### 6.1 Biochemical mechanisms involving HIE biomarkers

To date, the understanding of the potentially complex metabolic pathways and biochemical mechanisms of HIE is limited. According to current literature, it is a dynamic, multifactorial disease with a biochemical cascade caused by ischemia and hypoxia.^40^ The series of biochemical processes that occur during the progression of HIE mainly include the following: cellular bioenergetic exhaustion, excitotoxicity, loss of mitochondrial activity, oxidative stress (OS), and postischemic inflammatory response.^41^

### 6.2 Biomarkers for diagnosis and/or prognosis in the nonhuman primate HIE models

Many investigators have used metabolomics to identify changes in the content of various metabolites during the evolution of brain injury and during neuroprotective treatment in nonhuman primate models of ischemia and hypoxia, which can be used for HIE diagnosis and/or prognostic chemical markers.^42^, ^43^ One of the most pressing requirements for biomarker development in HIE is to assist in predicting long‐term outcomes, both to help inform clinicians and family members and to help better define pathways of care, including the transition to palliative care.^41^

HIE is mainly characterized by ischemia and hypoxia. Due to the reduction of blood flow, the blood supply of local tissue cells, especially brain tissue, is insufficient, resulting in energy failure, hypoxic acidosis, and brain damage in the body. In this case, the cells respire vigorously anaerobic and produce a large amount of lactate to compensate for the lack of oxygen and the consumption of ATP.^41^, ^44^ Elevated lactate levels in turn caused significant increases in metabolites from the glycolytic pathway and the tricarboxylic acid (TCA) cycle pathway. Among them, four TCA cycle intermediates, including citric acid, succinic acid, fumaric acid, and malic acid, showed significant increases after perinatal asphyxia. It is well known that the generation of ATP and other high‐energy phosphates is achieved through aerobic metabolic pathways, and the TCA cycle plays a key role in aerobic metabolism. Aerobic metabolism is essential for cellular activity, and energy storage, the required electron transport chain (located in mitochondria) combines with oxygen to generate more ATP through substrate phosphorylation, NADH, and FADH2. Infant transition from in‐utero to extrauterine life is also accompanied by metabolic changes in this TCA cycle intermediate.^25^, ^41^, ^45^ In the nonhuman primate model of HIE, the increase of citric acid, succinic acid, fumaric acid, and malic acid was abnormally significant, and the sharp increase of malic acid and succinic acid even exceeded that of lactic acid, which may be a more sensitive early diagnosis than lactic acid Indicator of birth asphyxia. At the same time, these data are consistent with the results from animal models of neonatal asphyxia, especially succinic acid and fumaric acid, two metabolites highly associated with asphyxia.^25^ On the other hand, succinic acid also plays a positive role. It has been proved that succinic acid is an extremely important angiogenic growth factor, and its action is mediated by G protein‐coupled receptor‐91 (GPR91).^25^, ^46^ In the retina, hypoxia leads to an increase in succinate, which massively activates GPR91 and promotes the production of vascular growth factors, including succinate. Maintaining a higher concentration of succinic acid in the brain is likely to play a role in cerebrovascular repair and healing after HIE in the brain.^25^, ^47^ In addition, the significant increase in arachidonic acid is due to the death of brain cells during brain injury, resulting in the leakage of fatty acids from the cell membrane after the cell membrane is ruptured. But what is interesting is that arachidonic acid has a dual effect on the central nervous system; on the one hand, it activates a syntaxin (STX‐3) involved in neuronal growth and repair.^25^, ^48^ On the other hand, it can act as a second messenger of lipids and regulate a variety of signaling molecules, among which phospholipase C (PLC)‐γ and δ, phosphokinase C (PKC)‐α and ‐β are inflammatory mediators and vasodilators.^47^ Therefore, the benefits and harms of arachidonic acid on the central nervous system cannot be weighed. More importantly, gamma‐butyric acid (GABA) is a substance. According to many pieces of literature, plasma GABA is a known marker of various metabolic and psychiatric diseases, and succinic acid and butyric acid are simultaneously produced during the catabolism of GABA; both acids are dramatically elevated in asphyxiated infants, so it is speculated that elevated levels of butyrate in asphyxiated infants reflect reduced hepatic degradation and/or the extent of impaired blood–brain barrier (BBB).^25^, ^49^, ^50^ Then there is propionic acid, which also shows an increasing trend in the HIE model. At present, it has been determined that it is mainly related to intestinal metabolism and is a potential biomarker of autism, but its related mechanism in brain ischemia is not clear.^25^, ^51^ What's more, from the research paper of Traudt. et al., they speculate that choline may be a biomarker and prognostic marker for the treatment of HIE. The reason is that nonhuman primates with HIE have the lowest choline levels after treatment.^52^

Notably, the study by Thomas R Wood and colleagues mentioned chemokines, 24 plasma cytokines, and growth factors as important plasma biomarkers for predicting the prognosis of neonatal HIE. Interleukin‐17 (IL‐17) and macrophage‐derived chemokine (MDC) were also identified as markers that distinguished normal/mild injury group from the moderate/severe injury group. IL‐17 at 6 h and MDC at 72 h were approximately 50% lower in the moderate/severe group than in the normal/mild group. IL‐17 is produced by the T‐cell lineage composed of cytokine 17 cells, T cells, and natural killer (NK) cells. MDC is a chemokine secreted by macrophages and dendritic cells and is associated with increased cell recruitment and inflammation. They also indicated that IL‐12p40 may be the best predictor of death or CP, with pro‐inflammatory signaling properties and the potential to prevent excessive inflammatory response, and was significantly reduced in the model group that died or had CP. However, modulation of IL‐12p40 levels has not been found to improve results after trials, so it is only a promising biomarker of injury, which has not been confirmed and needs to be studied.^26^

In summary, citric acid, succinic acid, fumaric acid, malic acid, arachidonic acid, GABA, IL‐17, MDC, IL‐12p40, and choline may be used as biomarkers for the diagnosis or prognosis of nonhuman primate HIE, which need more attention and energy to study and explore.

## 7 TREATMENT PROGRESS IN NONHUMAN PRIMATE MODELS OF HIE

With the development of medicine, more and more treatments and adjuvant treatments for HIE have been discovered, so the survival rate of premature infants with HIE is greatly improved. Here, this review summarized the therapeutic and adjuvant therapies that have been evaluated in studies in nonhuman primate models in recent years.

### 7.1 Respiratory therapy

In the absence of potentiating factors such as hypoxia, ischemia, or infection, premature delivery is associated with poor neonatal brain development and mild brain damage, which is generally well ameliorated by respiratory therapy. In the study by Loeliger et al., preterm nonhuman primates were maintained with positive airway pressure for 28 days after delivery: early continuous airway pressure or delayed continuous airway pressure, and brain growth and development were assessed histologically.^53^ It was found that although their brain weight and body weight were reduced, the brain/body weight ratio increased in preterm infants who were maintained on early continuous positive airway pressure (CPAP).^54^, ^55^ After positive pressure ventilation, although subcortical and deep white matter damage and gliosis still exist, both white matter damage and gliosis are significantly reduced. Early CPAP can reduce brain injury in preterm infants and can be used as an important adjuvant treatment for HIE.^53^ Some scholars have pointed out that inhalation of nitric oxide (NO) during CPAP can enhance ventilation in premature infants. Rees et al. reported that inhaled nitric oxide (iNO) may have a beneficial effect on the cortical gyrification and that iNO promotes the proliferation of astrocytes in the deep white matter.^56^, ^57^ Finally, they concluded that the protective effect of iNO on cortical development may be mediated through direct neuronal effects. But at the same time, they found that it seemed to exacerbate the destruction of the subarachnoid vasculature in some animals, forming hematomas.^58^ In conclusion, CPAP with NO inhalation may form subarachnoid hematoma, but iNO can enhance the therapeutic effect of CPAP on HIE, which is worthy of recognition.

### 7.2 TH

TH reduces the risk of death or severe neurodevelopmental disability in HIE to approximately 50%. TH can reduce ischemic hypoxic brain injury mainly by reducing the accumulation of excitotoxic neurotransmitters, brain metabolism, ATP depletion, lipid peroxidation of cell membrane, and release of oxygen and nitrogen free radicals. However, external treatment is still needed to further improve the brain injury.^52^ However, in the study of McAdams et al., the results were quite different from the previous results. In the study of nonhuman primate animal HIE models, TH did not improve the mortality and change the long‐term CP situation. They suspected this result is due to differences between nonhuman primates and humans, with cooling time being a primary consideration. Because primates age three times as fast as humans, the duration of 72 h of TH in nonhuman primate HIE models may already be beyond the window of benefit. Ultimately, they concluded that additional strategies are needed to mitigate long‐term neurodevelopmental damage in neonates with HIE.^22^

### 7.3 Erythropoietin (EPO) therapy

**Figure 3:**
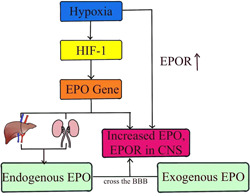
Production and delivery of erythropoietin (EPO): Hypoxia induces factor 1 (HIF‐1)‐triggered EPO gene expression, liver, and kidney secretion of EPO, and brain secretion of EPO, and upregulation of EPOR expression in the CNS. Brain damage caused by hypoxia increases the permeability of the blood–brain barrier (BBB), and EPO is more likely to cross the BBB and enter the brain to play its role. [Color figure can be viewed at wileyonlinelibrary.com]

Over the past three decades, considerable progress has been made in understanding the neuroprotective role of EPO, which has anti‐inflammatory, antioxidant, and neurorepair effects in preclinical studies of brain injury in term neonates.^26^ EPO is produced primarily in the kidney or liver of adults and in the liver of fetuses and newborn mammals. In addition, EPO can be induced to be produced in tissues other than the kidney and liver, such as the brain. EPO gene expression is triggered by hypoxia‐inducible factor 1 (HIF‐1), which is activated by various stressors, including hypoxia.^59^, ^60^, ^61^ Currently, EPO has been shown to be neuroprotective in several animal models of HIE. Christopher m. Traudt et al. reported that high‐dose EPO treatment reduced neuronal apoptosis, OL injury, inflammation, oxidative damage, NO toxicity, and glutamate toxicity, while increasing neurogenesis, oligodendrogenesis, glial cell proliferation, and angiogenesis.^52^ The study by Wood et al. explained that EPO has paracrine and autocrine functions in the brain. Following brain injury, EPO and its related receptors are upregulated by exposure to stimuli such as hypoxia and pro‐inflammatory cytokines. EPO binding promotes neurogenesis, angiogenesis, and oligodendrogenesis. Although EPO can promote the recovery of locally injured neuronal cells, it can also provide negative feedback to glial cells located in the penumbra, thereby limiting the scope of damage.^62^ The main function of EPO is mediated by its specific receptor (EPOR). There are two types of EPOR: a homodimer (EPOR/EPOR) that exists on erythrocyte progenitors, and a heterodimer and other cytokine receptors (EPOR/CD131) that exist on neurons and glial cells.^63^ In most cells, EPOR homodimers mediate hematopoietic responses, whereas heterodimer receptors mediate tissue protective activity.^64^ Most neurons probably express high levels of EPOR, and EPOR expression is upregulated in the presence of hypoxia, so Sanchez et al. proposed that EPO's action in the brain involves heterodimeric receptors.^65^, ^66^ EPO has a high molecular weight (30.4 kD), so the BBB through which EPO is transported in the brain is the main limiting factor for treatment. However, the brain injury caused by HIE will increase the permeability of BBB, so EPO can more easily cross the BBB during hypoxia, and the use of exogenous EPO has become a feasible method to treat HIE^67^ (Figure 3). At the same time, hypoxia also increases the transcription of endogenous EPO and EPOR in neurons or astrocytes, promoting the binding of EPO and EPOR.^68^ Above, accumulating data suggest that EPO should be included in these treatments. Therefore, the scientific basis and preclinical data for EPO neuroprotection are promising.

### 7.4 TH combined with EPO therapy

In recent years, many studies have proved that TH combined with EPO therapy will be safe and effective combination therapy. Traudt et al. showed that EPO + HT maintained cerebellar growth rate in animal models and improved many neurocognitive behavioral scores after perinatal asphyxia, which could improve the survival of neonatal patients with HIE and prevent the development of moderate to severe CP in a nonhuman primate model of perinatal asphyxia. They combined EPO therapy and TH to treat a nonhuman primate model of perinatal asphyxia and found no adverse events caused by Epo, suggesting that EPO is safe to use in the setting of HIE and HT. The combination of TH and EPO not only reduced the risk of death or moderate to severe CP to 0% but also maintained normal motor function.^22^, ^52^, ^69^ The study by Oorschot et al. also demonstrated that the combination of HT and EPO after neonatal HIE improved motor outcomes at 12 months of age compared with HT alone.^69^ In addition, Wood et al. showed that TH + Epo treatment increased monocyte chemotactic protein 4 (McP‐4) in animal plasma from 3 to 6 h and significantly decreased McP‐4 and MDC from 24 to 72 h, respectively. It has a significant neuroprotective effect.^26^ These studies lay the foundation for further studies on the role of TH+EPO in human neonates with HIE.

## 8 CONCLUSION

When it comes to studying serious diseases, nonhuman primates, which are anatomically similar to humans compared to other animal models, are the best choice for studying serious diseases. This review described the construction, behavioral and pathological manifestations of HIE models in nonhuman primates. It turns out that recent model construction, mainly clamping the umbilical cord to achieve perinatal asphyxia, is much finer than UCO models called Acute total asphyxia and partial asphyxia. The behavioral manifestations and brain pathology also differ between the two models, with partial asphyxia being more common and the pattern of brain injury more similar to the typical pattern of brain injury in human HIE. Among them, we should note that white matter injury is the most common pathological injury in nonhuman primate models and human HIE. Moreover, several studies have shown that nonhuman primates are highly similar to humans in terms of brain injury patterns and development. We also highlight a series of important biomarkers that can be used as diagnostic and/or prognostic indicators for HIE in a nonhuman primate neonatal HIE model. Finally, we further summarized the therapeutic approaches evaluated in nonhuman primate models in recent years and concluded that TH+EPO is currently the most effective approach in animal models. In my opinion, although, TH+EPO is currently the best, we should not stop to explore new and more perfect methods. In addition, there are many potential biomarkers that have not been confirmed, such as IL‐12P40, which need to be continuously studied, and we hope that these achievements can be successfully applied in clinical practice in the future.

## AUTHOR CONTRIBUTIONS

Yi‐Huan Guan completed the manuscript. Hong‐Su Zhou and Bo‐Yan Luo polished the whole article. Sajid Hussain and Liu‐Lin Xiong finalized and approved this paper. All authors have read and approved the final submitted manuscript.

## CONFLICT OF INTEREST STATEMENT

The authors declare no conflict of interest.

## TRANSPARENCY STATEMENT

The authors affirm that this manuscript is an honest, accurate, and transparent account of the study being reported; no important aspects of the study have been omitted; and any discrepancies from the study as planned (and, if relevant, registered) have been explained.

## ETHICAL APPROVAL

Not applicable.

## Data Availability

All data used in this review are available from the corresponding author upon request.
